# Dying on the Operating Table

**Published:** 1876-01

**Authors:** 


*Dying on the Operating Table.*—The Albany, New York, *Journal* says, on Thursday last Marcus H. Nelson, of Fort Ann, appeared at Dr. Armsby's office, in that city, and requested treatment for a finger which had been ulcerated a long time. It was pronounced gangrene, and he was sent to the hospital for treatment preparatory to the operation of amputation. On his admission to the hospital his weakened nervous condition indicated that he was apparently bordering on an attack of delirium tremens. On Friday, at the regular hospital clinic before the students, Dr. Armsby exhibited the case and remarked that he would defer the operation until the patient was in a better condition. The third finger of the right hand was black and gangrenous up to the second joint, and the patient was anxious and decided upon having the operation performed at once, affirming that if it was not done to-day he would go elsewhere, as he was determined to have it off: He was accordingly placed upon the operating table before the students, in the usual way, and a small quantity of ether and chloroform of equal parts given him. He inhaled freely, and in a short time manifested the usual stage of excitement, which was neither very severe nor long continued. In about three minutes he was pronounced ready for the operation, when it was discovered that he was pulseless. Immediately every effort was made to resuscitate him, without avail. Artificial respiration was continued for twenty minutes with no result. Dr. Shanks, house physician, affirmed that the quantity of ether and chloroform given was much less than on similar occasions, and the inhalation was in no way different from other cases.

